# Difference in the Prevalence of Elevated Blood Pressure and Hypertension by References in Korean Children and Adolescents

**DOI:** 10.3389/fmed.2022.793771

**Published:** 2022-02-24

**Authors:** Jeong Yeon Kim, Heeyeon Cho, Jae Hyun Kim

**Affiliations:** ^1^Department of Pediatrics, Samsung Medical Center, Sungkyunkwan University School of Medicine, Seoul, South Korea; ^2^Department of Pediatrics, Seoul National University Bundang Hospital, Seongnam, South Korea; ^3^Department of Pediatrics, Seoul National University College of Medicine, Seoul, South Korea

**Keywords:** hypertension, pediatrics - children, American Academy of Pediatrics (AAP), European society of hypertension (ESH), Korea (rep.), cardiometabolic risk

## Abstract

The prevalence of pediatric hypertension and obesity has increased over the past decades. Pediatric hypertension and obesity are associated with adult hypertension, metabolic syndrome, and cardiovascular disease. There are two main pediatric blood pressure (BP) classification guidelines, the “Clinical Practice Guideline for Screening and Management of High Blood Pressure in Children and Adolescents” (AAP 2017) and “2016 European Society of Hypertension guidelines for the management of high BP in children and adolescents” (ESH 2016). No study has classified Korean youths with cardiometabolic risk. This study analyzed the prevalence of high BP according to AAP 2017 (elevated BP and hypertension) and ESH 2016 (prehypertension and hypertension) in Korean children. Additionally, the cardiometabolic risk factors (CMRFs) were compared between children who were hypertensive in AAP 2017 but normotensive in ESH 2016 (upward reclassified) to those who were normotensive in both AAP 2017 and ESH 2016. Data were extracted from the Korea National Health and Nutrition Examination Survey, 2016–2018. A total of 1,858 children aged 10–17 years were included in the analysis. The prevalence of elevated BP/prehypertension and hypertension was 4.1 and 6.5% by ESH 2016, and 8.9 and 9.4% by AAP 2017 in Korean youth, respectively. The AAP 2017 reclassified 11.9% of youth in the upper BP class. When those upward reclassified youth were compared to those who were normotensive in both AAP 2017 and ESH 2016, reclassified youth were male predominant (77.1 vs. 49.6%, *p* < 0.001), older (14.6 vs. 13.8, *p* < 0.001) and showed higher body mass index (BMI) z-score (0.77 vs. 0.15, *p* < 0.001) and more overweight/obesity (14.0/30.6 vs. 13.3/6.4%, *p* < 0.001) and severe CMRFs (triglyceride 83.2 vs. 72.9 mg/dL, *p* = 0.011; high-density lipoprotein cholesterol 47.3 vs. 51.1 mg/dL, *p* < 0.001; alanine transaminase 21.7 IU/L vs. 14.7 IU/L, *p* = 0.001, uric acid 5.89 vs. 5.22 mg/dL, *p* < 0.001; metabolic syndrome 13.2 vs. 1%, *p* < 0.001). In conclusion, AAP 2017 showed a higher prevalence of abnormal BP in Korean youth, and those upward reclassified by AAP 2017 were more obese and had severe CMRFs than normotensive Korean children. The AAP 2017 could allow the early detection of Korean youth with more CMRFs.

## Introduction

Pediatric hypertension is a worldwide growing public health problem that is strongly associated with childhood obesity (1). Although the actual secular trend of hypertension prevalence varies from country to country, the evidence that childhood hypertension predisposes to adulthood hypertension, and metabolic syndrome (MS), and is the leading cause of adult mortality and cardiovascular disease (CVD), has highlighted the importance of recognizing pediatric hypertension (1–6). The diagnosis of pediatric hypertension is based on the normative blood pressure (BP) distribution measured by an auscultatory method in healthy children according to sex, age, and height.

Currently, there are two main updated pediatric hypertension guidelines; Clinical Practice Guideline for Screening and Management of High Blood Pressure in Children and Adolescents (AAP 2017) and 2016 European Society of Hypertension guidelines for the management of high blood pressure in children and adolescents (ESH 2016) (7, 8). The main differences between these two guidelines are: First, the BP classification criteria. Second, the inclusion (ESH 2016) and exclusion (AAP 2017) of overweight and obese children in normative reference to adjust for the impact of obesity on BP. Third, the age at which the adult hypertension definition applies (13-years-old for AAP 2017 vs. 16-years-old for ESH 2016) and the BP cut-off value in adults (9). As blood pressure differs according to race, ethnicity, and distribution of obesity, it is important to decide which guidelines should be applied locally (10). Most recently published studies have shown an increasing secular trend in hypertension prevalence and BMI in Korean children (11). The Korean Working Group of Pediatric Hypertension published a normotensive BP reference table excluding overweight and obese children based on data from the Korea National Health and Nutrition Examination Survey (KNHANES) 1998–2016 (12). However, a discussion on which BP classification criteria from the guidelines above should be applied to Korean children is still in progress. Several studies have compared cardiometabolic profile between AAP 2017 and ESH 2016 in other countries (13–17). Most studies showed better coverage of children with a cluster of cardiometabolic risks, such as hypertension in the AAP 2017 guideline (14, 15). This study analyzed the difference in hypertension prevalence and cardiometabolic risk profile between AAP 2017 and ESH 2016 in Korean youth using the KNHANES 2016–2018 data.

## Materials and Methods

### Study Participants

This study was performed using data obtained from the KNHANES 2016–2018. The KNHANES is a nationally representative survey that has been conducted cross-sectionally since 1998 by the Korea Disease Control and Prevention Agency (KDCA, formerly the Korea Centers for Disease Control and Prevention) and the Ministry of Health and Welfare. The detailed methods for the KNHANES data collection are described elsewhere (18).

A total of 24,269 individuals (11,071 males, 42.6%) were enrolled in the KNHANES from 2016 to 2018. The overall response rate for the survey was 76.6%. Among them, 1,858 children and adolescents aged 10–17 years were selected as participants for the present study, after excluding the following subjects: no anthropometric data (*n* = 130), no BP data (*n* = 134), diastolic BP values less than 30 mm Hg (*n* = 3), estimated glomerular filtration rate (GFR) by Schwartz equation <60 mL/min/1.73 m^2^ (19) (*n* = 0), or pregnancy (*n* = 0).

Among participants with normal BP according to ESH 2016 (*n* = 1,655), clinical data were compared between normal (*n* = 1,508) and abnormal (*n* = 147) BP by AAP 2017. Among them, in those with fasting blood samples (*n* = 1,454, normal BP 1,325, abnormal BP 129), subgroup analysis was performed to compare cardiometabolic risk factors (CMRFs) and MS.

The KNHANES protocol was approved by the Institutional Review Board of the KDCA. Informed consent was obtained from all the participants and/or legal guardian(s). The present study was approved by the Institutional Review Board of Seoul National University Bundang Hospital (IRB No. X-1906/547-903). All procedures were performed in accordance with the principles of Declaration of Helsinki.

### Measurement of Anthropometric and Laboratory Data

Anthropometric data were measured by well-trained medical personnel using calibrated equipment according to standardized protocols. Height was measured to the nearest 0.1 cm using a stadiometer (Seca 225, Seca, Hamburg, Germany). Weight was measured to the nearest 0.1 kg using an electronic balance (GL-6000–20, G-tech, Seoul, Korea). Body mass index (BMI) was calculated by dividing weight in kilograms by the square of height in meters. Waist circumference (WC) was measured to the nearest 0.1 cm using a flexible tape at the midpoint between the lowest costal margin and the uppermost tip of the iliac crest. Height and BMI were transformed to a standard deviation score (z-score) using the CDC 2000 Growth Chart (20, 21). Overweight and obesity was diagnosed when a participant's BMI was in the 85th to <95th percentile and the ≥95th percentile for corresponding sex and age, respectively. Abdominal obesity was defined when WC ≥90th percentile for corresponding sex and age (22).

Blood samples were drawn from participants by trained nurses after an overnight fast. Collected samples were properly prepared, transported to the Central Laboratory and analyzed within 24 h. Plasma glucose, high-density lipoprotein cholesterol (HDL-C), triglyceride, and alanine transaminase (ALT), and uric acid levels were measured using a Hitachi Automatic Analyzer 7600-210 (Hitachi, Tokyo, Japan). Glycated hemoglobin was measured using high-performance liquid chromatography (Tosoh, Tokyo, Japan), which is a certified method in the National Glycohemoglobin Standardization Program.

### Measurement of Blood Pressure and Definition of Hypertension

In the KNHANES, BP was measured using a mercury sphygmomanometer after resting for at least 5 min in a sitting position [Baumanometer Wall Unit 33(0850), W. A. Baum, New York, USA] with a cuff appropriate for arm circumference. All BP measurements were performed three times on the right arm. The average values of the second and third measurements of systolic and diastolic BP were used for subsequent analyses. Age-, sex-, and height-specific BP percentiles were determined for systolic and diastolic BP and classified as normal (<90th percentile), elevated BP (≥90th and <95th percentile), the hypertension (≥95th percentile) for age under 13 in AAP 2017 (8), and 16 in ESH 2016 guidelines (7). The static BP cut-off value was used to define normal, elevated BP, and hypertension for those older than these ages. (In AAP 2017, <120/ <80 mm Hg, 120/ <80 to 129/ <80 mm Hg, and ≥130/80 mm Hg, respectively. In ESH 2016, <130/85 mm Hg, 130–139/85–90 mm Hg, and ≥140/90 mm Hg, respectively). Among participants who were classified as normotensive by ESH 2016, those who are reclassified as hypertensive in AAP 2017 were described as “Upward reclassified,” and those who remained in normotension in AAP 2017 were described as “Persistent normotensive.” The high BP is defined when BP value is above normotensive.

### Definition of Metabolic Syndrome

MS was defined using criteria proposed by Cook et al. (23). The components of MS criteria are WC ≥ 90th percentile, fasting glucose ≥ 110 mg/dL, BP ≥ 90th percentile, triglycerides ≥ 110 mg/dL, HDL-C ≤ 40 mg/dL. Definition of MS is ≥ three among five criteria above mentioned.

### Statistical Analysis

Statistical analyses were performed using Stata 16.1 software (StataCorp LP, College Station, Texas, USA). A *svy* command with appropriate sample weights was used for the analysis. All data were expressed as weighted means with standard error for continuous variables and as the number of subjects with weight percentage for categorical variables. Student *t*-tests were used to compare continuous variables. Chi-squared tests were used to compare categorical variables. *P*-values <0.05 were considered statistically significant.

## Results

### Clinical Characteristics of Study Participants

Among the 1,858 participants, males were 972 (52.4%). Boys showed a higher height z-score, BMI z-score, proportion of overweight/obesity, WC, abdominal obesity, systolic BP and increased consumption of dietary factors (Table 1).

**Table 1:** Demographic characteristics of study participants.

**Variables**	**Total (*n* = 1,858)**	**Boys (*n* = 972, 52.4%)**	**Girls (*n* = 886, 47.6%)**	***P* value**
Age (year)	13.8 ± 0.1	13.8 ± 0.1	13.7 ± 0.1	0.402
Height z-score	0.39 ± 0.03	0.53 ± 0.04	0.23 ± 0.04	<0.001
BMI z-score	0.28 ± 0.03	0.40 ± 0.04	0.16 ± 0.04	<0.001
BMI category				
Obesity	209 (10.7%)	159 (15.3%)	50 (5.7%)	<0.001
Overweight	281 (14.4%)	165 (15.8%)	116 (12.7%)	
Waist circumference (cm)	69.7 ± 0.3	72.4 ± 0.4	66.8 ± 0.3	<0.001
Abdominal obesity	178 (9.0%)	147 (14.1%)	31 (3.4%)	<0.001
Systolic blood pressure (mm Hg)	108.1 ± 0.3	110.4 ± 0.4	105.6 ± 0.4	<0.001
Diastolic blood pressure (mm Hg)	66.1 ± 0.2	66.3 ± 0.3	66.0 ± 0.3	0.452
Dietary factors				
Calories (Kcal/d)	2100 ± 26	2339 ± 37	1838 ± 28	<0.001
Total fat (g/d)	57.6 ± 1.1	64.4 ± 1.7	50.1 ± 1.3	<0.001
Carbohydrates (g/d)	313.8 ± 4.1	346.7 ± 5.4	277.8 ± 4.6	<0.001
Protein (g/d)	76.5 ± 1.2	86.5 ± 1.7	65.6 ± 1.3	<0.001
Fiber (g/d)	20.5 ± 0.4	22.6 ± 0.5	18.1 ± 0.4	<0.001
Water (g/d)	773.7 ± 15.5	838.8 ± 21.8	702.2 ± 19.1	<0.001
Sodium (mg/d)	3071 ± 53	3473 ± 73	2630 ± 61	<0.001

*BMI, body mass index*.

*Data were expressed as weighted mean ± SE for continuous variables or number (weighted percent) for categorical variables*.

### Prevalence of Hypertension by Different Criteria

The prevalence of hypertension was 9.4% (boys, 11.1%; girls, 7.5%) by AAP 2017 and 4.1% (boys, 4.5%; girls, 3.5%) by ESH 2016 (Table 2). Boys showed higher prevalence of hypertension and elevated BP in AAP 2017.

**Table 2:** Prevalence of hypension by different criteria.

**Criteria**	**Category**	**Total**	**Boys**	**Girls**	***P*-value**
AAP 2017	<90th	81.7 (79.5, 83.7)	77.1 (73.8, 80.1)	86.7 (84.0, 89.0)	<0.001
	≥90th to <95th	8.9 (7.6, 10.6)	11.8 (9.6, 14.4)	5.8 (4.4, 7.7)	
	≥95th	9.4 (7.9, 11.2)	11.1 (8.9, 13.8)	7.5 (5.7, 9.8)	
ESH 2016	<90th	89.4 (87.6, 91.1)	89.1 (86.6, 91.3)	89.8 (87.3, 91.9)	0.637
	≥90th to <95th	6.5 (5.3, 7.9)	6.4 (4.8, 8.4)	6.6 (5.0, 8.6)	
	≥95th	4.1 (3.1, 5.3)	4.5 (3.2, 6.4)	3.5 (2.3, 5.5)	

*Data was expressed as weighted percent (95% CI)*.

*AAP 2017, the 2017 American Academy of Pediatrics Guideline; ESH 2016, the 2016 European Society of Hypertension Guideline*.

Among participants, 87.2% (boys, 82.9%; girls, 91.9%) showed the same BP category in both criteria (Table 3). A total of 11.9% (boys, 16.6%; girls, 6.8%) participants were underestimated using ESH 2016. In contrast, only 0.9% (boys 0.5%; girls, 1.2%) were overestimated using ESH 2016.

**Table 3:** Distribution of blood pressure of catetory by sex and criteria.

**Group**	**ESH 2016**	**AAP 2017**			***P*-value**
		** <90th**	**≥90th to <95th**	**≥95th**	
Total	<90th	80.9 (78.7, 83.0)	6.5 (5.3, 8.0)	2.0 (1.4, 2.9)	<0.001
	≥90th to <95th	0.8 (0.4, 1.4)	2.3 (1.7, 3.2)	3.4 (2.5, 4.6)	
	≥95th	0	0.1 (0.02, 0.4)	4.0 (3.0, 5.2)	
Boys	<90th	76.6 (73.3, 79.6)	10.0 (8.0, 12.4)	2.6 (1.6, 4.1)	<0.001
	≥90th to <95th	0.5 (0.2, 1.5)	1.8 (1.1, 2.9)	4.0 (2.7, 5.8)	
	≥95th	0	0	4.5 (3.2, 6.4)	
Girls	<90th	85.7 (82.8, 88.2)	2.7 (1.7, 4.3)	1.4 (0.8, 2.4)	<0.001
	≥90th to <95th	1.0 (0.5, 2.0)	2.9 (1.9, 4.3)	2.7 (1.8, 4.1)	
	≥95th	0	0.2 (0.05, 0.8)	3.3 (2.1, 5.3)	

*Data was expressed as weighted percent (95% CI)*.

*AAP 2017, the 2017 American Academy of Pediatrics Guideline; ESH 2016, the 2016 European Society of Hypertension Guideline*.

In non-obese youth, 90.6% (boys, 87.9%; girls, 93.2%) showed the same BP category in both criteria (Supplementary Table 1). A total of 8.5% (boys, 11.4%; girls, 5.9%) non-obese youth were underestimated using ESH 2016. In contrast, only 0.8% (boys 0.8%; girls, 0.8%) were overestimated using ESH 2016.

To analyze the CMRF distribution in high BP children according to AAP 2017 and ESH 2016, the prevalence of high BP in children with and without CMRF (overweight/obesity or metabolic abnormalities) was evaluated. A total of 1,619 participants with fasted samples were included. In AAP 2017, among 17.5% of high BP children, 52.0% had more than one CMRF. The prevalence of high BP in children with and without CMRF were 27.4% and 12.5%, respectively (*p* < 0.001). In ESH 2016, among 9.5% of high BP children, 58.3% had more than one CMRF. The prevalence of high BP in children with and without CMRF were 16.8% and 5.9%, respectively (*p* < 0.001).

### Comparison Between Upward Reclassified Youth and Persistent Normotensive Youth

Table 4 shows a comparison between upward reclassified youth and persistent normotensive youth. Among 1,655 subjects with normal BP by ESH 2016, 1,508 were persistent normotensive and 147 were upward reclassified. The upward reclassified youth showed male predominance, older age, higher BMI z-score, and more overweight/obesity. In the subgroup analysis, participants with fasting samples revealed elevated CMRFs including triglyceride, HDL-C, ALT, uric acid and MS.

**Table 4:** Comparison between subjects with normal blood pressure by ESH 2016 criteria.

	**Persistent normotensive (*n* = 1,508)**	**Upward reclassified (*n* = 147)**	***P*-value**
Sex, male, *n* (%)	744 (49.6%)	112 (77.1%)	<0.001
Age (year)	13.8 ± 0.1	14.6 ± 0.2	<0.001
Height z-score	0.37 ± 0.03	0.41 ± 0.08	0.641
BMI z-score	0.15 ± 0.03	0.77 ± 0.09	<0.001
BMI category			
Obesity	114 (6.4%)	41 (30.6%)	<0.001
Overweight	208 (13.3%)	24 (14.0%)	
Abdominal obesity	95 (5.4%)	35 (26.6%)	<0.001
Systolic blood pressure (mm Hg)	105.0 ± 0.2	120.0 ± 0.5	<0.001
Diastolic blood pressure (mm Hg)	64.4 ± 0.2	72.2 ± 0.7	<0.001
Estimated GFR (mL/min per 1.73 m^2^)	144.0 ± 0.8	144.1 ± 1.9	0.989
Glucose (mg/dL)^*^	91.4 ± 0.2	92.5 ± 0.7	0.140
HbA1c (%)^*^	5.35 ± 0.01	5.33 ± 0.02	0.381
Triglyceride (mg/dL)^*^	72.9 ± 1.2	83.2 ± 4.2	0.011
HDL-C (mg/dL)^*^	51.1 ± 0.3	47.3 ± 0.7	<0.001
Alanine transaminase (IU/L)^*^	14.7 ± 0.6	21.7 ± 2.0	0.001
Uric acid (mg/dL)^*^	5.22 ± 0.04	5.89 ± 0.14	<0.001
Metabolic syndrome, *n* (%)^*^	14 (1.0%)	16 (13.2%)	<0.001

*BMI, body mass index; HDL-C, high-density lipoprotein cholesterol*.

*Data were expressed as weighted mean ± SE for continuous variables or number (weighted percent) for categorical variables*.

* *n = 1,454 (normal 1,325, abnormal 129)*.

*Triglyceride and HDL-C were log-transformed for analysis and described as geometric mean ± SE*.

Comparable results were shown when 82 upward reclassified non-obese youth and 1,186 persistent normotensive non-obese youth were compared. The upward reclassified non-obese youth showed male predominance, older age, and had lower estimated GFR. In addition, higher fasting glucose, lower HDL-C, higher uric acid, and prevalent MS were shown in upward reclassified non-obese youth (Supplementary Table 2).

## Discussion

This study analyzed the prevalence of high BP according to the AAP 2017 (elevated BP and hypertension) and ESH 2016 (prehypertension and hypertension) guidelines in Korean children. We noted that the prevalence of hypertension was lower in Korea than in other countries from previous studies. In Korean children, approximately 12% in the AAP 2017 and 1% in the ESH 2016 were reclassified in the upper BP class. The AAP 2017 might potentially identify more children with prominent cardiometabolic risk factors.

In adults, hypertension is an important risk factor for CVD, the leading cause of death worldwide (23). Intensive control of hypertension can lower the risk of CVD (24). The trajectory phenomenon indicates that childhood BP influences adulthood BP emphasizing the importance of detecting hypertension in children who are at risk of developing CVD in future life to prevent adulthood hypertension and CVD (2–6, 25, 26). The cardiovascular effect of BP elevation in children is shown in subclinical findings such as left ventricular geometry changes and systolic and diastolic dysfunction (27–29). In children, improvement of left ventricular geometry changes has been shown after hypertension is treated with antihypertensive medication (30). Additionally, pediatric obesity is strongly associated with BP elevation in children and poor cardiovascular outcomes in adulthood (1, 31–33). When childhood obesity is adequately managed, CVD in adulthood also decreases (31).

There is no doubt that hypertension and obesity in childhood are risk factors for CVD in adults (2–6, 25, 26). However, as children scarcely present with actual CVD, the exact BP value that increases CVD is not known. Efforts have been made to define hypertensive children with cardiometabolic risk, and presently, there are two main pediatric hypertension guidelines, the AAP 2017 and ESH 2016 (7, 8). After the AAP 2017 was published, many studies compared the prevalence and cardiometabolic risk profile between the AAP 2017 and ESH 2016 to find adequate hypertension diagnosis criteria in children (13–17, 34). Pediatric BP is influenced by several factors, such as gender, obesity, nutrition, socioeconomic status, race, and ethnicity (10, 35). The AAP 2017 and ESH 2016 define hypertension primarily in children in the United States and Europe (7, 8). There are differences in influencing factors of BP between Western and Korean children, so the optimal guidelines for application in Korean children and adolescents are unknown. This study is the first comparative analysis of pediatric hypertension guidelines for Korean youth.

There were clinical differences by sex in the Korean pediatric population. Korean boys were taller and more obese and had higher systolic BP than girls. In addition, boys showed higher daily calorie, water, and sodium intake than girls. When the guidelines were applied to Korean children, elevated BP/pre-hypertension and hypertension prevalence increased from 6.5% to 8.9% and 4.1% to 9.4% by the AAP 2017 compared to ESH 2016. This study showed a lower prevalence of hypertension than that reported in previous studies from Spain (AAP 2017 vs. ESH 2016, 10.6% vs. 6.6%) (13) and China (AAP 2017 vs. ESH 2016, 8.3–14.5% vs. 5.0%) (16). Studies conducted in Italy showed a higher prevalence (AAP 2017 vs. ESH 2016, 31–35% vs. 35–41%), as these studies analyzed high-risk children who were overweight/obese or referred from primary physicians for BP elevation (14, 17). An increase in the prevalence of hypertension using the AAP 2017 guidelines was consistently observed in all the studies.

Statistically significant differences by gender were observed only in elevated BP (boys 11.8% vs. girls 5.8%) and hypertension (boys 11.1% vs. girls 7.5%) by the AAP 2017 in this study. However, ESH 2016 showed no gender differences in prevalence. Generally, boys tend to have higher BP values and more prevalent hypertension than girls (8, 36, 37). Besides this gender effect on BP, the clinical characteristics of Korean boys, higher obesity and high sodium intake, might have impacted the prevalence as only AAP 2017 showed gender differences. Obesity and high sodium intake strongly affect the prevalence of hypertension (33, 38). As CMRF effects on BP value, we also compared the prevalence of high BP in children with and without CMRF. Both AAP 2017 and ESH 2016 showed the more prevalent high BP in children with CMRF. In AAP 2017, the prevalence increased in both with and without CMRF children. These findings could raise the concern of overdiagnosis (false positive) by lowering the BP threshold in AAP 2017. However, CMRF is not the outcome of BP elevation. The clinical effects of these high BP children without CMRF need to be studied.

When these guidelines were compared in Korean children, ~12% and 1% were reclassified in the upper BP class in AAP 2017 and ESH 2016, respectively. A similar trend of a higher proportion of upward reclassification by AAP 2017 is shown in Italian overweight/obese children reported by Di Bonito et al. (15).

We compared children underestimated using ESH 2016 (upward reclassified by AAP 2017) to those consistently classified as normotensive in the AAP 2017 and ESH 2016. In a previous study by Di Bonito et al., upward-reclassified children were older, had higher BMI, higher insulin resistance, higher triglyceride, TC/HDL-C ratio, left ventricular (LV) mass index, and lower HDL-C compared to children steadily classified as normotensive in both guidelines (15). Similar to Di Bonito et al. (15), children reclassified upward by AAP 2017 in this study were more likely to be male, older, obese, and had poor metabolic factors (higher triglyceride, ALT, and uric acid; lower HDL-C; more prevalent MS) than those who remained normotensive by AAP 2017. These findings indicate that despite differences in race and ethnicity, applying AAP 2017 in Korean children still showed similar trends in Western children. In addition, AAP 2017 revealed more children with a cluster of cardiometabolic risks, such as hypertension. In other words, AAP 2017 might sensitively detect Korean youth who are obese and have other co-existing metabolic risks who were underdiagnosed by normotensive references derived from both normal and overweight/obese children. It provides public health opportunities to recognize Korean children with clusters of cardiometabolic risks, including obesity. In contrast to these findings, Antolini et al. reported there was no significant difference in detecting left ventricular hypertrophy between AAP 2017 and ESH 2016 when weight was adjusted within children who were obese or suspected to have BP elevation (17). Therefore, we analyzed the clinical difference of upward reclassified non-obese youth and persistent normotensive non-obese youth. Approximately 9% and 1% of non-obese youth were reclassified in the upper BP class in AAP 2017 and ESH 2016. Even after removing the impact of obesity, still upward reclassified youth showed poor CMRF. Globally, the prevalence of pediatric obesity has increased in the past decades, and a similar trend has been observed in Korean children (39–42). In the COVID-19 pandemic and lockdown era, children are more at risk of becoming obese and developing metabolic risks (43). Therefore, with the increasing obesity in Korean children, the AAP 2017 might potentially identify more children with prominent cardiometabolic risk factors.

This study has a strength because KNHANES is a national survey representing the Korean health and nutrition state, and this study presents the national prevalence of hypertension in children. Except for the study from China by Fan et al. (16), which analyzed hypertension prevalence in the national cohort, other previous studies compared the AAP 2017 and ESH 2016 in high-risk children or children referred for BP elevation (13–15, 17). This study is the first to evaluate cardiometabolic risk between AAP 2017 and ESH 2016 nationwide.

However, this study has some limitations. The KHANES has a cross-sectional design, and BP was measured only three times daily. Hypertension is diagnosed when BP elevation persists on more than three separate occasions, and when BP is measured in one event, it could overestimate the prevalence. Therefore, the actual prevalence of hypertension may be low. Additionally, no study has confirmed the causal relationship between pediatric hypertension and CVD in adulthood. We speculated that adult cardiometabolic risk is broadly known as subclinical surrogate markers such as obesity, MS, dyslipidemia, and LV geometry change (2–6, 25). Therefore, the surrogate markers discussed in this study might not be the actual risk of CVD development in later life. An ongoing cohort study evaluated the effect of pediatric hypertension on CVD (44). In the future, we might have a chance to determine whether our speculation is adequate.

In conclusion, this is the first study to compare the AAP 2017 and ESH 2016 in Korean children. The use of the AAP 2017 in Korean children may allow clinicians to diagnose hypertension in children with clusters of cardiometabolic risks and provide early intervention for risk control and prevention in the youth.

## Data Availability Statement

Publicly available datasets were analyzed in this study. All raw data used in the present study are available from the KNHANES webpage (https://knhanes.kdca.go.kr/knhanes/main.do) with the permission of the KDCA.

## Ethics Statement

The studies involving human participants were reviewed and approved by the Institutional Review Board of the Korea Disease Control and Prevention Agency and Seoul National University Bundang Hospital. Written informed consent to participate in this study was provided by the participants' legal guardian/next of kin.

## Author Contributions

HC and JHK contributed to conception and design of the study. JHK organized the database and performed the statistical analysis. JYK wrote the first draft of the manuscript. JYK, HC and JHK wrote sections of the manuscript. All authors contributed to manuscript revision, read, and approved the submitted version.

## Conflict of Interest

The authors declare that the research was conducted in the absence of any commercial or financial relationships that could be construed as a potential conflict of interest.

## Publisher's Note

All claims expressed in this article are solely those of the authors and do not necessarily represent those of their affiliated organizations, or those of the publisher, the editors and the reviewers. Any product that may be evaluated in this article, or claim that may be made by its manufacturer, is not guaranteed or endorsed by the publisher.

## References

[1] Song P Zhang Y Yu J Zha M Zhu Y Rahimi K. Global prevalence of hypertension in children: a systematic review and meta-analysis. JAMA Pediatr. (2019) 173:1154–63. 10.1001/jamapediatrics.2019.3310 31589252 PMC6784751

[2] Juhola J Oikonen M Magnussen CG Mikkilä V Siitonen N Jokinen E. Childhood physical, environmental, and genetic predictors of adult hypertension: the cardiovascular risk in young Finns study. Circulation. (2012) 126:402–9. 10.1161/circulationaha.111.085977 22718800

[3] Theodore RF Broadbent J Nagin D Ambler A Hogan S Ramrakha S. Childhood to early-midlife systolic blood pressure trajectories: early-life predictors, effect modifiers, and adult cardiovascular outcomes. Hypertension. (2015) 66:1108–15. 10.1161/hypertensionaha.115.05831 26558818 PMC4646716

[4] Yang L Magnussen CG Yang L Bovet P Xi B. Elevated blood pressure in childhood or adolescence and cardiovascular outcomes in adulthood: a systematic review. Hypertension. (2020) 75:948–55. 10.1161/hypertensionaha.119.14168 32114851

[5] Chen X Wang Y. Tracking of blood pressure from childhood to adulthood: a systematic review and meta-regression analysis. Circulation. (2008) 117:3171–80. 10.1161/circulationaha.107.730366 18559702 PMC3568631

[6] Juhola J Magnussen CG Viikari JS Kähönen M Hutri-Kähönen N Jula A. Tracking of serum lipid levels, blood pressure, and body mass index from childhood to adulthood: the crdiovascular risk in young Finns study. J Pediatr. (2011) 159:584–90. 10.1016/j.jpeds.2011.03.021 21514597

[7] Lurbe E Agabiti-Rosei E Cruickshank JK Dominiczak A Erdine S Hirth A. 2016 European society of hypertension guidelines for the management of high blood pressure in children and adolescents. J Hypertens. (2016) 34:1887–920. 10.1097/hjh.0000000000001039 27467768

[8] Flynn JT Kaelber DC Baker-Smith CM Blowey D Carroll AE Daniels SR. Clinical practice guideline for screening and management of high blood pressure in children and adolescents. Pediatrics. (2017) 140:e20171904. 10.1542/peds.2017-1904 28827377

[9] Brady TM Stefani-Glücksberg A Simonetti GD. Management of high blood pressure in children: similarities and differences between US and European guidelines. Pediatr Nephrol. (2019) 34:405–12. 10.1007/s00467-018-3946-y 29594504 PMC6162184

[10] Rao G. Diagnosis, epidemiology, and management of hypertension in children. Pediatrics. (2016) 138:e20153616. 10.1542/peds.2015-3616 27405770

[11] Cho H Kim JH. Secular trends in hypertension and elevated blood pressure among Korean children and adolescents in the Korea national health and nutrition examination survey 2007–2015. J Clin Hypertens. (2020) 22:590–7. 10.1111/jch.13842 32175671 PMC8029840

[12] Kim SH Park Y Song YH An HS Shin JI Oh JH. Blood pressure reference values for normal weight korean children and adolescents: data from the Korea national health and nutrition examination survey 1998–2016: the Korean working group of pediatric hypertension. Korean Circ J. (2019) 49:1167–80. 10.4070/kcj.2019.0075 31456368 PMC6875600

[13] Lurbe E Torró I Álvarez J Aguilar F Mancia G Redon J. Impact of ESH and AAP hypertension guidelines for children and adolescents on office and ambulatory blood pressure-based classifications. J Hypertens. (2019) 37:2414–21. 10.1097/hjh.0000000000002229 31688292

[14] Di Bonito P Valerio G Pacifico L Chiesa C Invitti C Morandi A. Impact of the 2017 blood pressure guidelines by the American academy of pediatrics in overweight/obese youth. J Hypertens. (2019) 37:732–8. 10.1097/hjh.0000000000001954 30817454

[15] Di Bonito P Licenziati MR Baroni MG Maffeis C Morandi A Manco M. The American academy of pediatrics hypertension guidelines identify obese youth at high cardiovascular risk among individuals non-hypertensive by the European society of hypertension guidelines. Eur J Prev Cardiol. (2020) 27:8–15. 10.1177/2047487319868326 31387383

[16] Fan H Zhang X. Difference in hypertension prevalence applying three childhood hypertension management guidelines in a national cohort study. J Hum Hypertens. (2020) 35:1038–45. 10.1038/s41371-020-00447-7 33239743

[17] Antolini L Giussani M Orlando A Nava E Valsecchi MG Parati G. Nomograms to identify elevated blood pressure values and left ventricular hypertrophy in a paediatric population: American academy of pediatrics clinical practice vs. fourth report/European society of hypertension guidelines. J Hypertens. (2019) 37:1213–22. 10.1097/hjh.0000000000002069 31022109

[18] Kweon S Kim Y Jang MJ Kim Y Kim K Choi S. Data resource profile: the Korea national health and nutrition examination survey (KNHANES). Int J Epidemiol. (2014) 43:69–77. 10.1093/ije/dyt228 24585853 PMC3937975

[19] Fadrowski JJ Neu AM Schwartz GJ Furth SL. Pediatric GFR estimating equations applied to adolescents in the general population. Clin J Am Soc Nephrol. (2011) 6:1427–35. 10.2215/CJN.06460710 21566103 PMC3109941

[20] Kuczmarski RJ Ogden CL Grummer-Strawn LM Flegal KM Guo SS Wei R. CDC growth charts: United States. Adv Data. (2000) 8:1–27. 11183293

[21] Kuczmarski RJ Ogden CL Guo SS Grummer-Strawn LM Flegal KM Mei Z. 2000 CDC growth charts for the United States: methods and development. Vital Health Stat. (2002) 11:1–190. 12043359

[22] Sharma AK Metzger DL Daymont C Hadjiyannakis S Rodd CJ LMS tables for waist-circumference and waist-height ratio Z-scores in children aged 5–19 y in NHANES III: association with cardio-metabolic risks. Pediatr Res. (2015) 78:723–9. 10.1038/pr.2015.160 26331767

[23] Cook S Weitzman M Auinger P Nguyen M Dietz WH. Prevalence of a metabolic syndrome phenotype in adolescents: findings from the third national health and nutrition examination survey, 1988–1994. Arch Pediatr Adolesc Med. (2003) 157:821–7. 10.1001/archpedi.157.8.821 12912790

[24] Yusuf S Joseph P Rangarajan S Islam S Mente A Hystad P. Modifiable risk factors, cardiovascular disease, and mortality in 155 722 individuals from 21 high-income, middle-income, and low-income countries (PURE): a prospective cohort study. Lancet. (2020) 395:795–808. 10.1016/s0140-6736(19)32008-2 31492503 PMC8006904

[25] Wright JT Jr Williamson JD Whelton PK Snyder JK Sink KM Rocco MV. A randomized trial of intensive vs. standard blood-pressure control. N Engl J Med. (2015) 373:2103–16. 10.1056/NEJMoa1511939 26551272 PMC4689591

[26] Sun SS Grave GD Siervogel RM Pickoff AA Arslanian SS Daniels SR. Systolic blood pressure in childhood predicts hypertension and metabolic syndrome later in life. Pediatrics. (2007) 119:237–46. 10.1542/peds.2006-2543 17272612

[27] Erlingsdottir A Indridason OS Thorvaldsson O Edvardsson VO. Blood pressure in children and target-organ damage later in life. Pediatr Nephrol. (2010) 25:323–8. 10.1007/s00467-009-1350-3 19946710

[28] Daniels SR Loggie JM Khoury P Kimball TR. Left ventricular geometry and severe left ventricular hypertrophy in children and adolescents with essential hypertension. Circulation. (1998) 97:1907–11. 10.1161/01.cir.97.19.1907 9609083

[29] Urbina EM Mendizábal B Becker RC Daniels SR Falkner BE Hamdani G. Association of blood pressure level with left ventricular mass in adolescents. Hypertension. (2019) 74:590–6. 10.1161/hypertensionaha.119.13027 31327264

[30] Tran AH Flynn JT Becker RC Daniels SR Falkner BE Ferguson M. Subclinical systolic and diastolic dysfunction is evident in youth with elevated blood pressure. Hypertension. (2020) 75:1551–6. 10.1161/hypertensionaha.119.14682 32362230 PMC7266265

[31] Sladowska-Kozłowska J Litwin M Niemirska A Wierzbicka A Wawer ZT Janas R. Change in left ventricular geometry during antihypertensive treatment in children with primary hypertension. Pediatr Nephrol. (2011) 26:2201–9. 10.1007/s00467-011-1916-8 21626453 PMC3203224

[32] Juonala M Magnussen CG Berenson GS Venn A Burns TL Sabin MA. Childhood adiposity, adult adiposity, and cardiovascular risk factors. N Engl J Med. (2011) 365:1876–85. 10.1056/NEJMoa1010112 22087679

[33] Franks PW Hanson RL Knowler WC Sievers ML Bennett PH Looker HC. Childhood obesity, other cardiovascular risk factors, and premature death. N Engl J Med. (2010) 362:485–93. 10.1056/NEJMoa0904130 20147714 PMC2958822

[34] Sorof J Daniels S. Obesity hypertension in children: a problem of epidemic proportions. Hypertension. (2002) 40:441–7. 10.1161/01.hyp.0000032940.33466.12 12364344

[35] Aksoy GK Yapar D Koyun NS Dogan ÇS. Comparison of ESHG (2016) and AAP (2017) hypertension guidelines in adolescents between the ages of 13 and 16: effect of body mass index on guidelines. Cardiol Young. (2021) 32:1–7. 10.1017/s1047951121003450 34420542

[36] Ewald DR Haldeman Ph DL. Risk factors in adolescent hypertension. Glob Pediatr Health. (2016) 3:2333794X15625159. 10.1177/2333794X15625159 27335997 PMC4784559

[37] Wang L Song L Liu B Zhang L Wu M Cao Z. Trends and status of the prevalence of elevated blood pressure in children and adolescents in china: a systematic review and meta-analysis. Curr Hypertens Rep. (2019) 21:88. 10.1007/s11906-019-0992-1 31599364

[38] Calcaterra V Larizza D De Silvestri A Albertini R Vinci F Regalbuto C. Gender-based differences in the clustering of metabolic syndrome factors in children and adolescents. J Pediatr Endocrinol Metab. (2020) 33:279–88. 10.1515/jpem-2019-0134 31927520

[39] Yang Q Zhang Z Kuklina EV Fang J Ayala C Hong Y. Sodium intake and blood pressure among US children and adolescents. Pediatrics. (2012) 130:611–9. 10.1542/peds.2011-3870 22987869 PMC9011362

[40] Di Cesare M Sorić M Bovet P Miranda JJ Bhutta Z Stevens GA. The epidemiological burden of obesity in childhood: a worldwide epidemic requiring urgent action. BMC Med. (2019) 17:212. 10.1186/s12916-019-1449-8 31760948 PMC6876113

[41] Rodriguez-Martinez A Zhou B Sophiea MK Bentham J Paciorek CJ Iurilli MLC. Height and body-mass index trajectories of school-aged children and adolescents from 1985 to 2019 in 200 countries and territories: a pooled analysis of 2181 population-based studies with 65 million participants. Lancet. (2020) 396:1511–24. 10.1016/s0140-6736(20)31859-6 33160572 PMC7658740

[42] Kim JH Moon JS. Secular trends in pediatric overweight and obesity in Korea. J Obes Metab Syndr. (2020) 29:12–7. 10.7570/jomes20002 32188238 PMC7118001

[43] Kim ES Kwon Y Choe YH Kim MJ. COVID-19-related school closing aggravate obesity and glucose intolerance in pediatric patients with obesity. Sci Rep. (2021) 11:5494. 10.1038/s41598-021-84766-w 33750841 PMC7943757

[44] Mendizábal B Urbina EM Becker R Daniels SR Falkner BE Hamdani G. SHIP-AHOY study of high blood pressure in pediatrics: adult hypertension onset in youth. Hypertension. (2018) 72:625–31. 10.1161/hypertensionaha.118.11434 29987102 PMC6202222

