# Supplementary material for: Difference in the Prevalence of Elevated Blood Pressure and Hypertension by References in Korean Children and Adolescents
Source: Front Med (Lausanne). 2022 Feb 24;9:793771. doi: 10.3389/fmed.2022.793771 (PMC8907724; doi:10.3389/fmed.2022.793771)
Supplement: Supplementary file 1 [file Table_1.docx]

Supplementary Table 1. Distribution of blood pressure of catetory by sex and criteria in non-obese youth.

| Group | ESH 2016 | AAP 2017 | | | P-value |
| --- | --- | --- | --- | --- | --- |
|  |  | <90^th^ | ≥90^th^ to <95th | ≥95^th^ |  |
| Total | <90^th^ | 86.7 (84.7, 88.5) | 4.6 (3.5, 5.9) | 1.7 (1.1, 2.7) | < 0.001 |
|  | ≥90^th^ to <95th | 0.7 (0.3, 1.5) | 1.7 (1.2, 2.6) | 2.2 (1.5, 3.3) |  |
|  | ≥95^th^ | 0 | 0.1 (0.03, 0.5) | 2.2 (1.4, 3.3) |  |
| Boys | <90^th^ | 85.0 (81.9, 87.7) | 6.8 (5.0, 9.1) | 2.4 (1.4, 4.2) | < 0.001 |
|  | ≥90^th^ to <95th | 0.8 (0.3, 2.2) | 0.7 (0.3, 1.6) | 2.2 (1.3, 3.9) |  |
|  | ≥95^th^ | 0 | 0 | 2.2 (1.3, 3.9) |  |
| Girls | <90^th^ | 88.3 (85.4, 90.6) | 2.5 (1.5, 4.4) | 1.1 (0.6, 2.1) | < 0.001 |
|  | ≥90^th^ to <95th | 0.6 (0.2, 1.9) | 2.7 (1.8, 4.3) | 2.3 (1.5, 3.7) |  |
|  | ≥95^th^ | 0 | 0.2 (0.06, 1.0) | 2.2 (1.1, 4.0) |  |

Data was expressed as weighted percent (95% CI)

Abbreviations: AAP 2017, the 2017 American Academy of Pediatrics Guideline; ESH 2016, the 2016 European Society of Hypertension Guideline.

Supplementary Table 2. Comparison between subjects with normal blood pressure by ESH 2016 criteria in non-obese youth

|  | Persistent Normotensive  (n = 1186) | Upward Reclassified  (n = 82) | P-value |
| --- | --- | --- | --- |
| Sex, male, n (%) | 549 (47.2%) | 58 (70.0%) | <0.001 |
| Age (year) | 13.9 ± 0.1 | 14.6 ± 0.3 | 0.013 |
| Height z-score | 0.27 ± 0.03 | 0.33 ± 0.12 | 0.560 |
| BMI z-score | -0.18 ± 0.03 | -0.02 ± 0.09 | 0.106 |
| Abdominal obesity | 2 (0.2%) | 0 (0%) | 0.206 |
| Systolic blood pressure (mm Hg) | 104.5 ± 0.2 | 119.1 ± 0.7 | <0.001 |
| Diastolic blood pressure (mm Hg) | 64.3 ± 0.3 | 72.6 ± 0.9 | <0.001 |
| Estimated GFR (mL/min per 1.73m^2^) | 143.5 ± 0.9 | 139.7 ± 1.8 | 0.043 |
| Glucose (mg/dL)* | 90.9 ± 0.3 | 92.8 ± 0.9 | 0.041 |
| HbA1c (%)* | 5.34 ± 0.01 | 5.30 ± 0.03 | 0.133 |
| Triglyceride (mg/dL)* | 69.4 ± 1.2 | 71.0 ± 4.9 | 0.740 |
| HDL-C (mg/dL)* | 52.01 ± 0.4 | 49.9 ± 1.0 | 0.030 |
| Alanine transaminase (IU/L)* | 13.1 ± 0.5 | 12.8 ± 0.6 | 0.741 |
| Uric acid (mg/dL)* | 5.07 ± 0.04 | 5.55 ± 0.18 | 0.006 |
| Metabolic syndrome, n (%)* | 0 (0%) | 2 (2.5%) | <0.001 |

Abbreviations: BMI, body mass index; HDL-C, high-density lipoprotein cholesterol.

Data were expressed as weighted mean ± SE for continuous variables or number (weighted percent) for categorical variables.

*n=1454 (normal 1325, abnormal 129)

Triglyceride and HDL-C were log-transformed for analysis and described as geometric mean ± SE.
